# Abstracts DGPRÄC

**DOI:** 10.1515/iss-2019-2007

**Published:** 2019-03-20

## DGPRÄC/DGU: Interdisciplinary concepts for treating difficult injuries of the lower extremities

### Challenges of reconstruction in plastic, aesthetic and reconstructive Surgery or the constant battle for blood supply – From David to Goliath

(Abstract ID: 552)

A. Limbourg^1^, P. M. Vogt^1^

^1^*Medizinische Hochschule Hannover*

**Background:**

Reconstruction in plastic surgery comprises a vast field, with varying levels of demand. The vascular territories (angiosomes) oft the body define the options of micro- or macro tissue transplantation. Differential challenges arise from supermicrosurgical approaches with reformation of aesthetic units over smart and effective solutions for medium size defects by perforator flaps up to complex approaches with arterialized flaps and their vascular extension by loops in case of tremendous trauma and large size defects. Whereas supermicrosurgical reconstructions requires miniture skill in anastomosing fine vessles. Experience and fantasy of the surgeon achieves effective coverage of medium size defects by perforator flap approaches. The resurrection from large defects, however, as in polytrauma patients, requires many prerequisits to lead to success. First and foremost thourough planning, e.g. loop- and flap preformation, awarness of caveats that comes with experience and importantly high end interdisciplinarity and intesive care excellence to achieve survival and durable results.

**Materials and methods:**

We investigated the defects and surgical strategies of a series of patients with different degrees of defects ranging from facial defects over smaller size defects with smart perforator flap solutions up to coverage of extreme defects of trauma which are considered mutually exclusive with life. Next to the surgical approach and conduct we put emphasis on the blood supply situation, severity of accompanying disease, such as organ transplant and immunosupression, metabolic disease and excessive and life-threatening trauma that compromize the vascular tree.

**Results:**

We present a case of a patient with facial recosntruction using a miniaturized ALT-flap under the aggravating circumstances of pharmacological immunosupression und therapeutic anticoagulation with problems of wound healing and bleeding. We introduce our solution to cover small pretibial defects by differential positioning of both rotor blades of a propeller-flap of the lower extremity to cover the involved defects simultanously. Finally we demonstrate the stepwise approach to coverage of a tremendous 40 x 50 cm life threatening defect oft the back with osteosynthetic exposure following severe pressure trauma.

**Conclusion:**

The differential challenges in plastic surgery arise from the location and size oft the defect but also from limitations oft the vascular territories as well as the medical circumstances and condition of a patient. With our case series we demonstrate plastic surgical solutions to defect coverage from supermicrosurgical, aesthetically relevant approaches over durable solutions of medium size problems up to excessively large and life-threatening defects of severe trauma.

## DGPRÄC: 3D Evaluation for preparation of plastic surgery operations

### Virtual Reality in preoperative planning

(Abstract ID: 241)

A. K. Bartella^1^, I. Bartella^2^, A. Pabst^3^, F. Hölzle^1^, I. Scholl^4^, J. Steegmann^1^, B. Lethaus^1^

^1^*Uniklinik RWTH Aachen*

^2^*Uniklinik Köln*

^3^*Bundeswehrkrankenkenhaus Koblenz*

^4^*FH Aachen, Aachen*

**Background:**

Not only that current imaging modalities (CBCT, CT, MRI) are becoming more precise in capturing data, also the demonstration and interpretation of the acquired images is no longer limited to conventional display screens or projectors. The so-called Virtual Reality (VR) glasses have the potential to engage the viewer in a three-dimensional (3D) space and ultimately evaluating the reconstructed anatomical structures from a novel new perspective.

**Materials and methods:**

For the first time in the field of Oral and Maxillofacial Surgery (OMFS), a 3-dimensional imaging data set (CBCT, CT, MRI) is evaluated by using VR glasses. A medical student, an OMFS resident and an OMFS consultant rated the preoperative usability of VR glasses to improve the operative understanding for three patient cases, namely a deeply impacted wisdom tooth, a midfacial fracture, and an oncological resection case.

**Results:**

VR glasses seem to help in simplify operations and give the surgeon a good preoperative overview of the intraoperative findings especially in the evaluation of impacted teeth and hard tissue structures. In addition, this technique represents a promising innovative modality for training of surgical residents in addition to student teaching. The more experienced the surgeon is, the lower is the additional value of VR glasses is.

**Conclusion:**

The preoperative examination using VR glasses can aid in a better understanding and planning of the surgical site in the future, and represents an innovative advanced technology for displaying CT, CBCT, and MRI anatomical data sets.

### Step by Step Guide to Ultrasound Based Design of Perforator Flaps by the Microsurgeon – Device Settings, Basic and Advanced Application

(Abstract ID: 415)

A. Kehrer^1^, N. Sachanandani^2^, N. Platz Batista da Silva^3^, S. Geis^1^, E.-M. Jung^3^, J. P. Hong^4^, L. Prantl^1^

^1^*Universitätsklinikum Regensburg*

^2^*Banner M.D. Anderson Cancer Center, Gilbert*

^3^*Universitätsklinikum Regensburg*

^4^*Asan Medical Center, Seoul*

**Background:**

Perforator flaps have become a popular option in reconstructive tissue transfers. Consistent with the "hot/cold zone" concept for rapid dissection and thin flap harvest, reliable pre-operative perforator mapping is mandatory. Systematic review of the literature has demonstrated color-coded duplex sonography (CCDS) to have the highest pooled sensitivity and positive predictive valueto identify perforating vessels. The question remains: why has CCDS for micro vessel mapping not been more widely applied by microsurgeons? The following presentation of a step-by-step guide reviews the following aspects: 1. probe selection and device settings 2. structured mapping approach 3. pedicle position planning 4. safe flap design 5. prediction of perforator course 6. advanced application. Decisive practical steps are demonstrated with a patient series.

**Materials and methods:**

Experiences with ultrasound-guided flap design gained from perforator flap free tissue transfers performed 7/2013-3/2019, without using other technology, was the basis of our guide. Our structured method comprises standardized markings, patient positioning, and simple ergonomics. Basic CCDS pre-settings, selection, and conventional probe guidance are outlined for the microsurgeon. Easy orientation through different tissue layers and framing of micro vessels in color duplex mode are described. Power Doppler and B-flow mode may be added to enhance sensitivity for small perforators. Pulse Wave (PW)-mode aids in perforator selection.

**Results:**

Linear multifrequency probes (6 to 15 MHz) were used for perforator detection.Favorable device properties are depth of focus set to 3-5 cm, color gain set low enough to reduce extravascular color signals, wall filter (WF) low,and pulse repetition frequency (PRF) low to 0.5-20 Hz. Preset programs facilitate settings. A 100% concordance rate was seen comparing pre-operative perforator visualization with CCDS and intraoperative findings. CCDS proved to be easy to learn for the microsurgeon, inexpensive, convenient, and highly accurate. Picture and video material is demonstrated to illustrate tissue appearance and perforator characteristics.

**Conclusion:**

CCDS is a powerful tool for the microsurgeon to perform preoperative micro vessels mapping and evaluation in perforator flaps.

### 3-D Volumetric Acquisition of small volume changes of the face

(Abstract ID: 709)

T. L. Schenck^1^

^1^*Klinikum der Universität München, LMU München*

**Background:**

Soft-tissue filler injections for the treatment of facial aging can result in different skin surface effects depending on the targeted facial fat compartment and fascial plane. This work investigates the tissue response of defined amounts of injected soft-tissue filler material into superficial and deep facial fat compartments via the calculation of the surface-volume coefficient.

**Materials and methods:**

Four fresh frozen cephalic specimens obtained from human donors (3 female, 1 male; mean age 74.96 ± 22.6 years; mean BMI 21.82 ± 6.3 kg/m2) were studied. The superficial and deep lateral forehead compartments, deep temporal fat pad, sub-orbicularis oculi fat compartment and the deep medial cheek fat compartment were injected with aliquots of 0.1cc of contrast enhanced material and scanned using 3D surface imaging.

**Results:**

The sub-orbicularis oculi fat compartment revealed the highest correlation coefficient (rp=0.992, p<0.001) and the highest surface-volume coefficient (svc=0.94). The compartment with the lowest tissue response was the deep medial cheek fat compartment (rp=0.745, p<0.001; svc=0.29), followed by the deep lateral forehead compartment (rp=0.814, p<0.001; svc=0.68), superficial lateral forehead compartment (rp=0.824, p<0.001; svc=0.74), and the deep temporal fat pad (rp=0.947, p<0.001; overall svc=0.64).

**Conclusion:**

These results, confirmatory in their nature to current injection strategies, provide evidence for the validity and reliability of the surface-volume coefficient. Injection procedures should be targeted in terms offacial fat compartments and fascial planes for a desired aesthetic outcome as each fat compartment and fascial plane has unique tissue responses to injected soft-tissue fillers.

## DGPRÄC: Up-to-date treatment approaches of vascular wounds, decubitus and necrotising fasciitis

### The thoracodorsal artery perforator (TDAP) flap as an alternative for the surgical treatment of axillary acne inversa

(Abstract ID: 458)

C. Varnava^1^, S. Stenske^1^, N. Münstermann^1^, N. Gebur^1^, D. Kampshoff^1^, M. Kückelhaus^1^, P. Wiebringhaus^1^, T. Hirsch^1^

^1^*Fachklinik Hornheide, Münster*

**Background:**

The axillary acne inversa is a common skin disease that often requires a surgical approach.

In many cases, appropriate therapy can be provided through excision of the entire sweat-gland-bearing area. The resulting defects pose a challenge to the plastic surgeon due to the localization and the regularly severely scarred surrounding tissue. Usually, local flaps or skin grafts are applied to achieve defect coverage.

**Materials and methods:**

In this case, we demonstrate soft tissue reconstruction with a TDAP flap in a patient with postoperative scarring and movement restriction following resection of axillary acne inversa and secondary wound healing.

**Results:**

There were no major complications in wound healing. The Shoulder was functional and the movement was not restricted when compared with the opposite side.

**Conclusion:**

We discuss postoperative outcome after excisional therapy and secondary wound healing versus immediate axillary soft tissue reconstruction using pedicled thoracodorsal-artery perforator (TDAP) flaps regarding OR Time, functional and aesthetic outcome.

According to our experience the TDAP flap is an excellent alternative for the immediate coverage of axillary defects after resection of acne inversa.

Advantages are the reduced donor site morbitity, the wide arc of rotation and the delicate skin texture. Disadvantages might be extended operating time.

### Necrotising fasciitis – LRINEC-Score, modern surgical management and other new aspects to an old problem: increasing survival in a deadly disease

(Abstract ID: 601)

C. Koch^1^, D. Bitzinger^1^, S. Klein^1^, C. Taeger^1^, A. Anker^1^, B. Graf^1^, L. Prantl^1^, A. Kehrer^1^

^1^*Universitätsklinikum Regensburg*

**Background:**

Necrotising fasciitis (NF) is a live threatening infection involving the deep fascia and subcutaneous tissue. Without surgery it is marked by rapid progression with high lethality. Differential diagnosis with other soft tissue infections is often difficult and delayed. Clinical signs vary from reddening, swelling and unproportionate pain.

NF can be classified into three categories, which respect localization disease and triggering bacteria. The Laboratory Risk Indicator for Necrotizing Fasciitis (LRINEC)-Score has been described for differentiation.

The aim of this study is to develop an algorithm by laboratory parameters, radiological imaging, histopathological findings and clinical signs to quickly confirm the correct diagnosis and consequently indicate an appropriate surgical treatment. Goals were limb salvage, function and survival.

**Materials and methods:**

Between the 2008 and 2018 cases of NF at the Department of Plastic-, Hand- and Reconstructive Surgery at the University Hospital of Regensburg were included. Our retrospective study examined the value of LRINEC, finger test and other clinical signs to diagnose and assess the extent of the disease. Surgical interventions and plastic reconstructions are evaluated.

**Results:**

A total of forty-two patients could be included. All cases required plastic-surgical interventions by serial debridement followed by soft tissue reconstruction.

A guiding algorithm including new parameters as the LRINEC-Score, finger test and more signs is presented. LRINEC-score is a laboratory test for necrotizing fasciitis including the parameters Haemoglobin, Leucocytes, Sodium, Creatinin, Glucose and CRP. It has proven its value for our cohort.

The finger-test may show a weak bleeding and putride secretion after incision of the skin and subcutaneous tissue. If digital splitting of the deep fascia is possible the test result is positive.

Leading clinical symptoms include early signs by fever, hypotension, erythema, tachypnoea or tachycardia, edema and limited vigilance.

Late signs comprise hemorrhagic bullae of the skin, crepitations of subcutaneous soft tissue or muscles, dark blue to black skin coloration, skin necrosis and multi organ failure. Patient examples are illustrated.

**Conclusion:**

Early diagnosis of NF helps to save live and limb. In our cohort the value of clinical signs like finger test and lab diagnostics like the LRINEC-Score could be confirmed. Early interdisciplinary treatment by intensive care medicine, plastic surgery and other disciplines is mandatory to increase survival. Instant and consequent debridements show good results in stopping progressive spread of disease as seen in secondary explorations. Soft tissue reconstruction improves function.

### Complication management of a highly critical disease: Purpura Fulminans and Implications for surgery

(Abstract ID: 609)

C. Koch^1^, S. Geis^1^, C. Hirche^2^, J. Dolderer^1^, L. Prantl^1^, A. Kehrer^1^

^1^*Universitätsklinikum Regensburg*

^2^*BG Unfallklinik Ludwigshafen*

**Background:**

Purpura fulminans (PF) is a critical disease caused by meningococcal septicaemia mostly in childhood. It is characterized by high lethality, extensive necroses and mutilations of extremities. Other ethiologies are idiopathic forms or purpura neonatorum, which is characterized by deficiency of protein C. PF is triggered by micro-embolism of the vascular system, followed by quickly spreading necroses of skin and different organs. Modern concepts of intensive care treatment in the acute phase of the disease and early surgical interventions lead to a rising number of surviving patients requiring limb salvage. Aim of this study is to evaluate a two-center case series to define determining factors for lower morbidity, improved extremity salvage and better function through early surgical interventions.

**Materials and methods:**

Between 1998 and 2018 patients with PF at two centers for critical wound care, the department of plastic-, hand- and reconstructive surgery of the university hospital of Regensburg and the Department of Plastic Surgery, BG-Clinic Ludwigshafen, Heidelberg University, Germany were included into our study.

Our retrospective study examined patients with PF, which were stabilized by intensive care medicine and received different surgical interventions. After survival of the acute phase patients received plastic surgical reconstructions.

**Results:**

A total number of eight patients could be included. All patients survived the first phase. All cases required plastic-surgical interventions, because of extensive loss of skin and soft tissue. Flap reconstruction was judged as necessary in five cases. Hereby four defects could be reconstructed with free tissue transfer, one by a local flap. Flap survival was at 100 percent. One case needed revision of arterial anastomosis.

Cases that received early debridements including consequent fasciotomies and secondary plastic-surgical reconstructions rendered good functional results. Limb salvage was achieved in three patients. One patient died because of the fulminant progress of the disease.

**Conclusion:**

Management of PF requires a multidisciplinary approach and close communication between the different subspecialties. Early debridements with consequent fasciotomies showed good results in salvaging subfascial muscle tissue in the extremities with satisfying functional results. Early surgical interventions is a key factor for improved limb salvage and survival.

## DGPRÄC: Hand endoprosthetics 

### Implementing free neurovascular toe-(joint)-transfers as a reconstructive procedure for amputation injuries of two and tripartite fingers with substance loss

(Abstract ID: 652)

E. Brix^1^, S. Geis^1^, C. Taeger^1^, S. Engelmann^1^, C. Wenzel^1^, J. Dolderer^1^, L. Prantl^1^, A. Kehrer^1^

^1^*Universitätsklinikum Regensburg*

**Background:**

Complex injuries of the hand are often accompanied by loss of essential functional features of the phalanges. Common reconstructive procedures for minor defects in the distal third of the thumb are Moberg-, Cross-Finger- or Littler flaps. Medium sized soft tissue defects may be reconstructed with Foucher-flaps. Other homo- or heterodigital flap procedures are viable for coverage of tripartite phalanxes. Free flap transplantations complete this arsenal of operative reconstructive procedures. Microneurovascular toe transfers allow for most detailed reconstructions. Our experiences and practical knowledge with free toe transplants are reported and diversely discussed with respect to contending procedures in the following study.

**Materials and methods:**

From 2010 until 2018 12 partial or complete toe transfers were carried out at the University Medical Center Regensburg. Juxtaposed was a control group of patients treated with contending reconstructive procedures.

**Results:**

A total of 4 neurovascular pulp transplantations and 8 osteocutaneous- partial (2), complete (2), as well as vascularized toe joint (4) transfers were carried out. Nine flaps healed without complications, one pulp transfer demonstrated inadequate blood perfusion but was maintained as a composite graft. All toe transplant healed without loss of length in the phalanges. Range of motion, remaining length and resensitization have proven to be superior in free toe transplants opposed to the control group treated with contending methods.

**Conclusion:**

In our heterogenic patient collective free toe transplants have proven to be superior for functional reconstruction of two and tripartite phalanxes opposed to common local reconstructive procedures. However, their indication should follow a strict protocol. Finger pulp regions can be substituted by homogenous sensitive ridged skin, lost joints may be replaced by autologous neojoints. Donor site morbidity was moderate.

## DGPRÄC: Extremity reconstruction in burn surgery 

### Microsurgical reconstruction of the Plantar Foot: Long term Functional Outcomes and quality of life

(Abstract ID: 77)

P. Heidekrueger^1^, P. Prantl^1^, A. Thiha^1^, M. Ninkovic^2^, N. Broer^2^

^1^*Universitätsklinikum Regensburg*

^2^*Klinikum Bogenhausen, München*

**Background:**

When faced with plantar defects, reconstruction of the weight-bearing areas presents unique surgical challenges. Several free flap modalities have been described in this respect, but there remains debate regarding the best suited flap modality. Aim of this study is to compare free muscle and non-neurotized fasciocutaneous flaps for plantar reconstruction in respect to long-term functional outcomes.

**Materials and methods:**

Overall, 89 patients received 100 free flaps (ALT n=46; gracilis n=54) for plantar reconstruction. The data were screened for patients’ demographics, as well as perioperative details. Postoperative complications were accounted for and the two groups compared accordingly. All patients were contacted for a long-term follow-up examination.

**Results:**

There were no significant differences between the two groups regarding major- (24 versus 17 percent; p=0.366) and minor surgical complications (61 versus 70 percent; p=0.318). However, the ALT group showed a significantly higher need for secondary surgeries (39 versus 19 percent; p=0.022). 68 patients (76 percent) returned for long-term follow-up evaluation (mean 51.2 months, range 13-71 months). The ALT group showed significantly less pain at the recipient- (p=0.0004) and donor (p=0.010) sites and scar assessment revealed significantly better results (p<0.001). Additionally, the ALT group showed better depth- (p=0.017) and superficial (p=0.007) sensation and enabled better shoe provision (p=0.014).

**Conclusion:**

Both, the free ALT- and gracilis flaps are well suited for plantar reconstruction, yielding overall similar functional outcomes. However, the ALT flap produces less scarring and pain, while showing better recovery of sensation and enabling better shoe provision. The ALT-flap thus presents our preferred option.

## DGPRÄC: Body lift after significant weight loss

### Circumferential lower body lift with auto-buttock augmentation: A new approach

(Abstract ID: 764)

T. S. Peltz^1^

^1^*Prince of Wales Hospital, Sydney*

**Background:**

With continuously rising BMIs in our society and the growing accessibility of patients to bariatric surgery body lift procedures are becoming more and more common. These contouring surgeries are invasive and complicated surgical interventions. We want to present a new simple staging concept for the surgical contouring of the lower body and describe our method of auto-augmenting the gluteal region in a circumferential body lift.

**Materials and methods:**

All in all 41 patients underwent a circumferential lower body lift procedure in the last 3 years. 25 patients underwent the procedures without auto augmentation of the gluteal region and 16 patients were operated including an auto-augmentation of the buttock area. To augment the buttock area a modified perforator flap technique was applied "SGA perforator rotation flap". Results of both groups were compared regarding operating time, complication rates, overall result. Cosmetic results were analysed using conventional standardized photography and 3D scanning. The scans were performed with an high resolution Artec 3D scanner pre and post surgery.

**Results:**

A significant improvement of buttock contouring can be achieved with this operating method. Aesthetic results can be individualised to patient’s wishes/expectations: -by flap design (shape, width, length, thickness) -by pocket dissection (shape, width, depth) -by flap fixation (sutures, infra-gluteal fold reconstruction (lower pole)). Complication rates were not higher in the augmentation group when compared to the conventional body lift group.

**Conclusion:**

The auto-augmentation of the gluteal region in a body lift procedure via “SGA perforator rotation flap” is safe, reliable and very effective technique to overcome the undesired flat buttock problem accompanied with conventional lower body lift procedures. 3D scanning is an objective method to compare and improve techniques in body contouring surgery.

**Picture:**
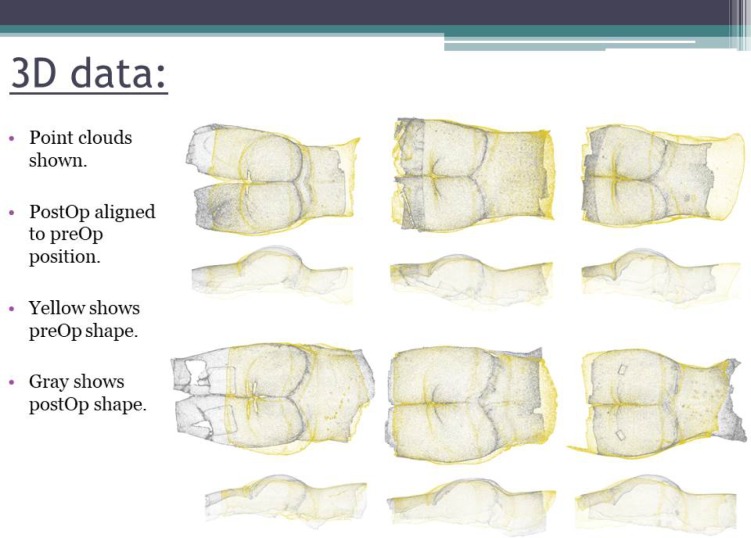
3D example files of LBL patients

## DGPRÄC: Peripheral nerve surgery

### Axon Analysis in Human Cranial Nerves – Quick and Simplified Semi-automatic Computer-Based Axon Quantification Method in Context of Facial Reanimation

(Abstract ID: 403)

S. Engelmann^1^, M. Rüwe^1^, V. Mandlik^1^, S. Geis^1^, E. Tamm^2^, R. Bleys^1^, L. Prantl^1^, A. Kehrer^1^

^1^*Universitätsklinikum Regensburg*

^2^*Universität Regensburg*

**Background:**

Microscopic analysis of peripheral nerves is key to many clinical and basic science research projects. In the past, no prime and uniform method could be found that is simple, cost-efficient, and time-sparing. For small research collectives, manual analysis has been used. Attempts have been made by several research groups throughout medical and scientific communities to automate this process. These methods are often highly specialized and therefore only accessible to a few specialized laboratories. Within our facial nerve study, it was necessary to create an efficient, cost-effective method for axon quantification, due to the large number of 1238 nerve biopsies examined.

**Materials and methods:**

A total of 106 cadaveric facial halves were dissected, up to 19 extracranial specimens of the facial and masseteric nerve were biopsied. Slides were histologically prepared with PPD staining and 200x magnified transections were digitized using a microscope (Zeiss Imager Z1) with a mounted camera (Zeiss Axio cam MR). A refined method for semi-automatic axon counting was developed aiming to combine the accuracy and low-cost of manual counts with the speed of automated morphometry using widely available Fiji freeware. This method combined several processing and analyzing steps into one macro so that multiple images can be processed instantly (Figure 1). Manual counts were obtained using a count and click method with the cell counter function in Fiji freeware. A statistical comparison between the novel method and manual counts was carried out using 129 randomly selected biopsies from a study cohort of 1238 (10.4%).

**Results:**

Semi-automatic axon counting took 1 hour and 47 minutes for all 129 biopsies (average 50 sec per biopsy). The counting process is automatic and must not be accompanied. Manual counting took 21 hours and 6 minutes in total (average 9 minutes and 49 seconds per biopsy). The novel technique shows a linear correlation to the manual method (R=0.944 Spearman rho). The semi-automatic method to find nerve biopsies with >900 axons was found to be sensitive (94%) and specific (87%).

**Conclusion:**

The novel semi-automatic technique proved to be very time-sparing compared to the manual counting method. In absolute axon counts there is a loss of accuracy within larger nerve biopsies. However, the cut-off value of 900 can be determined reliably. Therefore, we suggest our user-friendly, cost-effective and time-efficient method can be suggested as an alternative in microscopic peripheral nerve morphometry in anatomic studies considering axon margins. Based on precision validation its applicability may be enlarged to other cranial and peripheral nerves.

**Picture:**
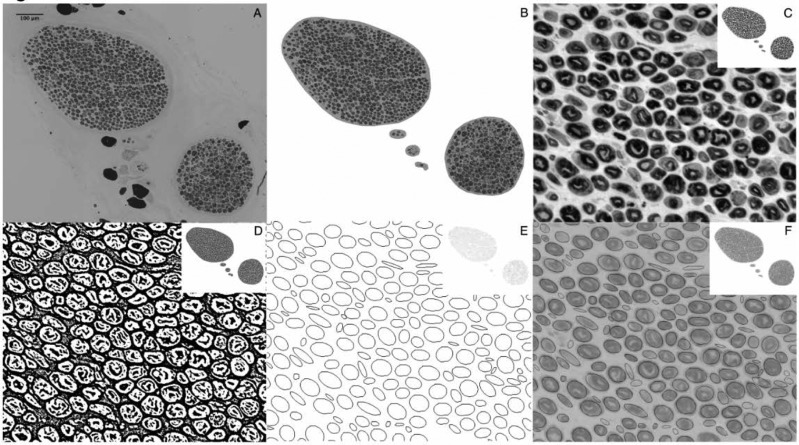
Figure 1. Automated Macro Sequence in single Steps: A) Grey scale image, cranial zygomatic branch, 200x magnified, unprocessed. B) Fascicles extracted (Background and artefact deletion) C) Contrast enhancement with Fijis CLAHE Local contrast enhancement function D) Auto Local Threshold binary image E) Analyze Particles F) Overlay for demonstration purposes.

### Correlation of axonal capacity with diameter of facial nerve branches relevant for facial reanimation: Nerve morphometry in 106 hemifaces

(Abstract ID: 469)

M. Rüwe^1^, S. Engelmann^1^, V. Mandlik^1^, C. Taeger^1^, E. Tamm^2^, R. Bleys^3^, L. Prantl^1^, A. Kehrer^1^

^1^*Universitätsklinikum Regensburg*

^2^*Universität Regensburg*

^3^*University Medical Center, Utrecht*

**Background:**

Peripheral facial palsy causes severe functional, aesthetic and psychological impairments. Restoration of function and facial expressions is possible through cross-face-nerve-grafts (CFNG) and functional muscle transplantation. Various criteria must be taken into account to choose the right donor nerve for coaptation. Previous studies have shown that an axon capacity exceeding 900 axons is correlated with good functional results. The aim of our study is to correlate axon numbers with the diameter of zygomatic and buccal facial nerve branches to facilitate donor nerve selection.

**Materials and methods:**

Antegrade microsurgical dissection was performed on 106 hemifaces of fresh unpreserved cadavers. Nerve biopsies were taken in clinically important donor nerve regions of the zygomatic and buccal system. Level I and level II branches were classified as relevant for CFNG coaptation. Nerves were PPD-fixed, sectioned, and stained for digital semi-automated axon quantification. Cross sections were measured by two orthogonal vectors using Zeiss AxioVision software which was first calibrated with a micrometer scale. Nerves in situ were presumed to be round. The measurement included nerve sheath structures and perineurium comparable to a clinical setting.

**Results:**

A total number of 788 branches were evaluated. Quality standards for semi-automated axon analysis and diameter measurement were met by 495 specimens. A diameter of one millimeter correlated with 1834 ± 693 axons (n= 68; r= 0.66; p= 0.0001) in major zygomatic branches and for major buccal branches 1851 ± 913 axons (n= 58; r= 0.37; p= 0.004). In downstream zygomatic branches 1067 ± 531 axons / mm (n= 161; r= 0.61; p= 0.0001) could be found. Downstream buccal branches showed 1208 ± 530 axons / mm (n= 208; r= 0.58; p= 0.0001). Axonal density in the buccal system was consequently higher than in the zygomatic system (p= 0.006).

**Conclusion:**

Overall axon density decreased from the facial main trunk to peripheral branches. In order to achieve a cut-off value of 900 axons, donor nerve branches in the zygomatic system are required to demonstrate a diameter >0.84 mm, whereas in buccal branches >0.74 mm.

### Microanatomic Study on 106 Cadaveric Facial Halves – Axonal Load of Important Donor Nerve Branches for Facial Reanimation by Cross-Face-Nerve-Grafting

(Abstract ID: 641)

S. Engelmann^1^, M. Rüwe^1^, V. Mandlik^1^, S. Geis^1^, E. Tamm^2^, R. Bleys^3^, L. Prantl^1^, A. Kehrer^1^

^1^*Universitätsklinikum Regensburg*

^2^*Universität Regensburg*

^3^*University Medical Center Utrecht*

**Background:**

Patients with irreversible facial palsy experience emotional and functional distress. In the past, different concepts have evolved to ameliorate facial symmetry and function by using Cross-face-nerve-grafting (CFNG) and transplantation of functional muscles. A correlation between the effect of axonal load of donor nerves on the functional and aesthetic outcome of CFNGs for facial reanimation was described in literature, defining a cut-off of >900 axons to achieve good functional outcomes. A common requirement for successful facial reanimation procedures is an excellent topographic and microanatomic knowledge. The primary aim of this study is a comprehensive presentation of respective details of the facial nerve’s micro- and macroanatomy to improve surgical practice in facial reanimation.

**Materials and methods:**

A total of 106 facial halves of fresh frozen cadavers were dissected under 4x loupe magnification. The extracranial course of the facial nerve was exposed in anatomical dissection. Nerve biopsies were obtained at up to 15 defined clinically relevant anatomical locations. Specimens were fixed, histologically sectioned and digitized at 200x magnification for semi-automatic analysis to determine axonal load via a self-improved method using Fiji freeware.

**Results:**

A total of 1084 biopsies were acquired. After quality control, 955 could be processed. The main facial nerve trunk showed an axon capacity of 6684 ± 1884 (2655 to 12457). Peripheral nerve branches of the zygomatic and buccal system showed a range from 58 to 3370 and 42 to 5726, respectively. Largest axonal loads were found in the two most caudal zygomatic branches with 1030 ±840 and 1128 ±519 and the two most cranial buccal branches with 1040 ±705 and 1006 ±819. Temporal and marginal mandibular branches were also analyzed showing 1191 ±668 and 1603 ±849 axons, respectively. A schematic orientation system is provided for all facial branches.

**Conclusion:**

Axonal load within one anatomic location is quite variable. This study gives a precise microanatomic insight to potential donor nerve branches and the entirety of the relevant facial nerve branching system. It was shown that caudal zygomatic and cranial buccal branches demonstrated the largest axonal loads in average. Proximal buccal and zygomatic, as well as temporal and marginal mandibular main branches exceed the cut-off axonal load of 900 axons reliably. In facial nerve surgery, our findings along with clinical parameters such as nerve thickness, function, and intraoperative electrostimulation must be considered.

## DGPRÄC: Sehnenverletzungen der Hand - von der Primärverletzung zur Rekonstruktion (in Kooperation mit der DGH)

### Rupture rate, functional outcome and patient satisfaction after primary flexor tendon repair using the Arthrex FiberLoop^®^ and Tsuge suture technique with early active motion rehabilitation

(Abstract ID: 630)

S. V. Koehler^1^, F. Neubrech^1^, M. Sauerbier^1^

^1^*BG Unfallklinik Frankfurt am Main*

**Background:**

While the current literature in flexor tendon repair primarily supports a 4-strand locking core suture and epitendinous repair, the optimal suture material remains unclear. Identifying the ideal suture material could help provide sufficient stability for early dynamic splint therapy and thus improve total active motion (TAM), grip strength and daily activities. We hypothesize the rupture rate after flexor tendon repair with the Arthrex FiberLoop® (Arthrex, Munich, Germany) is lower than other suture materials and functional outcome and patient satisfaction are superior compared to the current literature.

**Materials and methods:**

A 2-stage retrospective, randomized follow-up study of 143 patients treated with the Arthrex FiberLoop® after flexor tendon injury in zones 2 or 3 from May 2013 - May 2017 was performed. In the 1st stage, the rupture rate in all patients was assessed after a follow-up of >1 year to exclude revision surgery. In the 2nd stage, 20% patients (29 patients) were randomly clinically examined. Functional parameters, such as finger and wrist mobility measured by goniometer, grip strength measured by Jamar dynamometer, patient satisfaction measured by school grades (1-6), pain-levels measured by visual analogue scales (0-10), and DASH-score were assessed. The Buck-Gramcko and Strickland scores were calculated to compare our results to the current literature.

**Results:**

A rupture rate of 2.1% was recorded. A postoperative complication was reduced TAM. 29 patients (20% of all patients) were examined at a mean of 34 ±7.5 months postoperatively. 10.3% of these patients had an incomplete fingertip palmar distance. Postoperative grip strength was on average 24 ±3.1 kg. 93.0% of these patients were very satisfied with treatment. No patient complained of pain postoperatively. The mean postoperative DASH score was 6.7 ±2.8 points. The mean Buck-Gramcko score was 14 ±0.2 points. According to the Strickland score, 93.0% patients had excellent and 6.99% good results.Patients returned to work at a mean of 4 ±0.7 months postoperatively.

**Conclusion:**

Primary flexor tendon repair with the Arthrex FiberLoop® has proven to be a viable treatment option. It leads to acceptable pain relief and grip strength and most ADL’s can be maintained. Although there are process-specific complications, patient satisfaction remains high. Our rupture rate with the Arthrex FiberLoop® is lower than other suture materials and our Buck-Gramcko and Strickland scores are superior compared to the current literature.

### A biopolymer adhesive film for sutureless epitendinous repairs: An ex vivo comparison in the porcine deep flexor tendon model

(Abstract ID: 760)

T. Peltz^1^

^1^*Prince of Wales Hospital, Sydney*

**Background:**

Biopolymer adhesives have recently emerged as an alternative for joining of tissues in various fields of surgery. This study investigates the use of a novel chitosan based adhesive biopolymer foil for the use of tendon repairs.

**Materials and methods:**

In an ex vivo laboratory experiment porcine deep flexor tendons were repaired by either:

Group 1. Simple circumferential suture epitendinous repair + Adelaide core repair (current gold standard)

Group 2. Sutureless biopolymer adhesive foil + Adelaide core repair

In both groups a Cross Locked Cruciate (Adelaide) core tendon repair (4/0 Ticron) was used. In group one an additional simple circumferential epitendinous repair (6/0 Prolene) was used. In group 2 no circumferential suture repair was used. Instead, a chitosan biopolymer film (2.5 x 2.5 cm) was wrapped around the porcine tendon and activated by laser-activation from an infrared diode laser (GaAIAs diode). Both sample groups were tested using an Instron-5543 (Canton, USA) tensile testing machine. Endpoints were tensile strength, maximum load and load at clinical failure (2mm gapping force).

**Results:**

Sutureless biopolymer supported Adelaide repairs are easier and faster to perform. The biopolymer repaired tendon provided higher load to failure stability when compared to conventional circumferential suture supported Adelaide repairs.

**Conclusion:**

In this first preliminary laboratory study biopolymer supported Adelaide repairs could show superior mechanical stability in mechanical testing szenarios. The repairs are easier to reproduce and faster to perform.

Activated biopolymer films could present an alternative for conventional sutured circumferential tendon repairs in the near future.

**Picture:**
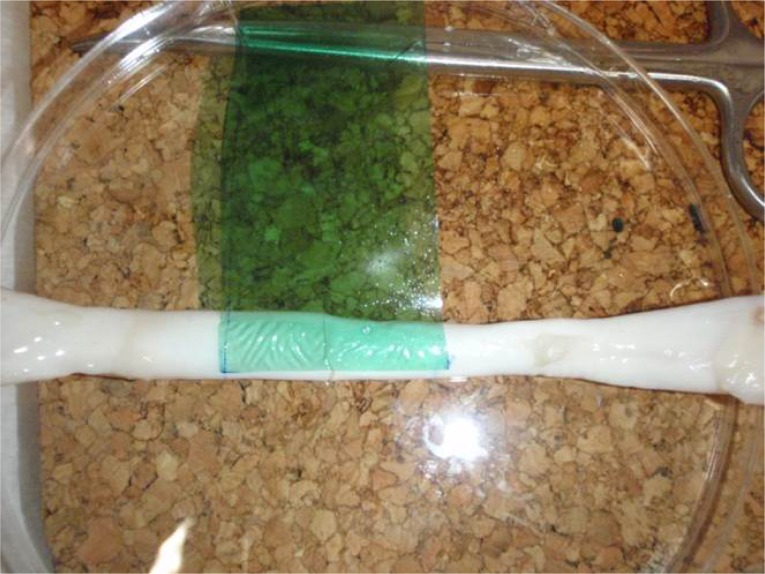
Chitosan based bioadhesive applied to tendon

### Tendon repair training: What is the best approach to teach tendon reconstructions?

(Abstract ID: 763)

T. S. Peltz^1^

^1^*Prince of Wales Hospital, Sydney*

**Background:**

Laceration of a flexor tendon is a common injury. Most tendon repairs are performed by junior residents/registrars in emergency settings. Training courses for young surgeons to learn tendon repair surgery techniques are limited and there is no consensus as to which is the best model for teaching.

**Materials and methods:**

Training of tendon repair surgery can be taught in 4 steps: 1. Theory 2. Practical training on artificial cotton rolls 3. Practical training on isolated ex-vivo tendons 4. Practical training on ex-vivo but in-situ deep flexor tendons in zone II in pig trotters or turkey feet.

**Results:**

This build-up of training steps guarantees teaching of the ability to perform a four strand Adelaide suturing technique in the isolated tendon and the adequate handling and appreciation of the anatomy and complicated arrangement of tissues in this area. In the last step turkey feet are preferred since the anatomical comparability from the human hand to the turkey foot is more accurate than to the pig trotter.

**Conclusion:**

Tendon repair surgery should be trained in a laboratory setting before surgeons perform repairs in clinical settings. Cadaveric hands are difficult to obtain and ethical implications need to considered. A combination of training on artificial cotton rolls, isolated animal tendons and pig trotters / turkey feet is ideal and should be anticipated to achieve maximum learning progress for young surgeons. We present a tendon repair course program using artificial cotton rolls, pig tendons and turkey feet.

**Picture:**
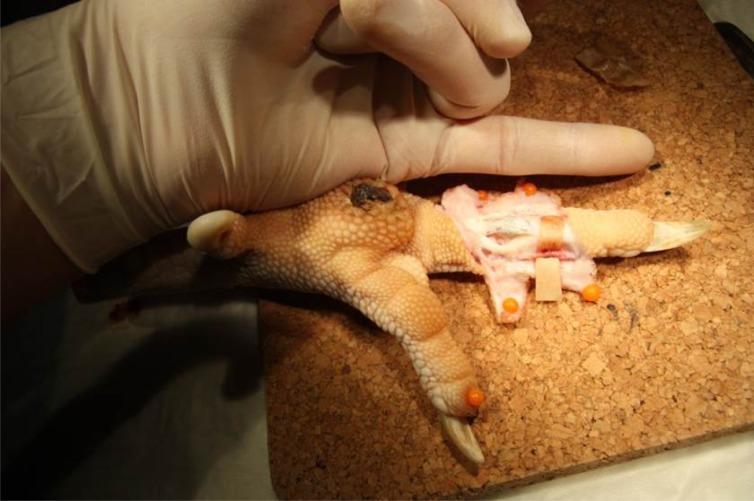
Anatomy of the flexor apparatus in the turkey foot

